# Visualizing patterns and gaps in transgender sexual and reproductive health: A bibliometric and content analysis of literature (1990–2020)

**DOI:** 10.1080/26895269.2021.1997691

**Published:** 2021-11-30

**Authors:** Liam Gary Arnull, Anuj Kapilashrami, Margaret Sampson

**Affiliations:** a Institution of Population Health Sciences, Queen Mary University of London, London, United Kingdom; b School of Health and Social Care, University of Essex, Essex, United Kingdom; c Library Services, Children’s Hospital of Eastern Ontario (CHEO), Ottawa, Ontario, Canada

**Keywords:** Bibliometric analysis, content analysis, health, human rights, reproductive justice, sexual and reproductive health, transgender

## Abstract

**Background:**

Transgender people face numerous obstacles to accessing adequate, affordable, and appropriate sexual and reproductive health (SRH) services as outlined by the UN’s Sustainable Development Goal 3.7 target of achieving universal access to SRH services by 2030. However, transgender SRH sits as a poorly researched area within public health that makes it difficult to understand the current dilemmas facing transgender SRH. This article reports the findings of a study aimed at taking stock of global research in transgender SRH.

**Methods:**

A bibliometric analysis, used to gain insights from the retrieved literature’s metadata, alongside a content analysis were utilized to examine the growth, impact, and content of retrieved articles.

**Results:**

Nine hundred fourteen journal articles were retrieved, predominately in English (884; 96.7%). These involved 3653 authors from 46 affiliated countries. Most frequent keywords included HIV, PrEP, and gender identity; corresponding to the SRH issues studied, namely HIV/AIDs and gender reassignment. Top cited and overall articles originated heavily from US affiliated authors. Content analysis outlined the articles’ inclusion of the transgender community to largely have a mixed focus with cisgender people in research, these articles largely disease-focused and conducted within cities in the United States.

**Conclusions:**

Growth in transgender SRH research was minimal until the early-2010s, after which a steep rise can be observed. Research retrieved has a disproportionate clinical and biomedical focus around HIV and related STI issues suggesting a failure to engage with reproductive justice and more comprehensive rights-based understanding of SRH. The sustained use of derogative language suggests a need for greater inclusion and awareness of trans identities within research and publishing. The dominance of the United States in authorship and as a site of research establishes the need for more geographically diverse research, trans, and LMIC-led research enquiry and creating greater opportunities for cross-cultural, comparative, and collaborative scholarly work.

## Background

Transgender or trans people face significant challenges to accessing healthcare, encountering hurdles often unseen by cisgender people (Moseson et al., 2020; Ross et al., 2021; Strauss et al., 2021). Throughout trans sexual and reproductive health (SRH) care, sustained hetero-cis-normativity (Worthen, 2016), transphobia, and poor awareness and lack of training for health-workers often render crucial health services unapproachable (Gridley et al., 2016; Luvuno, Ncama, & Mchunu, 2019; Reisner et al., 2016).

A bibliometric analysis conducted by Sweileh (2018) offered preliminary but useful insight into literature surrounding transgender health, outlining barriers, and limitations within current discourse. These included limited publications around transgender health from Africa and the Mediterranean, focus on mental health research, and a rise in publications from 2005 (pp. 6–7).

Their work’s broad focus on the whole of transgender health, however, restricts the ability to examine the interactions and impacts of transgender SRH issues, specifically as patterns are generalized. This article sets out to deepen this enquiry by auditing the current state of knowledge on trans SRH, outlining what is studied by whom and identifying key gaps and future areas of research. Using software unused by Sweileh, Biblioshiny, and additional exploration via content analysis on retrieved documents, this work will also generate new insights into the impacts and relations of literature around transgender SRH.

SRH sits as a critical aspect within the healthcare and well-being of trans individuals (Koch, McLachlan, Victor, Westcott, & Yager, 2020). Globally trans people face fragmented health provision and legal protections (Castro-Peraza et al., 2019; Reisner et al., 2016) with the United Nations (UN) Office of the High Commissioner for Human Rights in 2020 noting the alienation of trans people that prevents “them from enjoying their sexual and reproductive health and rights” (OHCHR, 2020, p. 1). The UN itself has outlined the need for universal access to sexual and reproductive services by 2030 (UNDP, 2021).

SRH continues to be a critical challenge for the transgender community with the World Health Organization (WHO) recognizing that “addressing the health inequities defined by gender identity might well be a test of our ability to leave no one behind while achieving the SDGs [Sustainable Development Goals]” (Thomas et al., 2017, p. 5). However, limited research exists into the needs and experiences of transgender and non-binary SRH, limiting planners’ knowledge of these issues (Wanta & Unger, 2017). To better understand this, then, our research question centers on exploring: “what impact, relationships, and insights does the current body of literature around transgender sexual and reproductive health provide?”

## Methods

### Bibliometric analysis

A bibliometric analysis is a quantitative exploration of metadata that accompanies published literature, such as citation details, year of publication, and author locations. This allows analysis around article growth, relationships between authors and their institutions, and developing insights into issues examined via keyword usage. It also provides a review of the impact that the retrieved literature has had in terms of article and journal citation numbers (Durieux & Gevenois, 2010; Iftikhar et al., 2019). This analysis, then, works toward our research objective by providing a review of where the literature has gone so far whilst also outlining subsequent gaps in research – allowing space to discuss the implications of this alongside future steps that can be taken to advance this body of knowledge.

To achieve this, the following steps were taken:

1. First, different indexing databases were reviewed (e.g., PubMed, Web of Science, etc.) with Scopus being decided upon to undertake a literature search due to its large database of retrieved articles and multidisciplinary nature – allowing for a broad range of literature to be drawn from (AlRyalat, Malkawi, & Momani, 2019; Gasparyan, Ayvazyan, & Kitas, 2013; Sweileh, 2018).
2. Then, we developed a search strategy (presented in the Supporting Information) to systematically capture literature dealing with transgender SRH. This involved reviewing keywords from relevant studies, experimenting with new keywords and searcher operators to optimize the relevance and precision of retrieved literature, and then shaping this into a search strategy through relevant search logic operators (e.g., AND, OR, etc.). Sweileh’s search strategy (2018) was used as a baseline for keywords that were built upon and updated by drawing on recent scholarship on trans terminologies (Gender Minorities Aotearoa, 2016; Shopland & Leeworthy, 2018; Vincent, 2018) to better capture literature around the transgender community and more comprehensively retrieve studies on SRH. For instance, terms such as “genderflux” were added to better increase the retrieval of trans literature or further developing Sweileh’s terms such as “two-spirits,” to increase its flexibility and sensitivity within the search strategy^1^ – enabling the retrieval of trans related studies that might otherwise be missed. As the indexing of documents can be imperfect, this search strategy departed from Sweileh’s focus on retrieving literature through excluding non-health subject areas and instead developed specific terms for SRH to retrieve documents via keywords. Relying on defined keywords to capture SRH within the text of the documents works to diminish the retrieval of unwanted data.
3. Using this search strategy, an initial search was carried out that found limited articles present before 1990, around 0 to 2 publications per year, so the search period was subsequently defined between 1990 and 2020.
4. From this, the bibliometric data that accompanies this defined set of literature was exported through the download options provided by Scopus. Bibliometric data is the metadata that is indexed alongside documents found in index databases, such as citation counts and funding details. The articles’ title and abstract were reviewed for relevance and the overall search strategy was refined by adding new exclusions or adjusting the logic functions to ensure only relevant literature was retrieved.
5. Next, a final export from Scopus was downloaded and cleaned for duplicate/empty entries, as not all bibliometric data entries are perfectly populated. An archive of this search export can be found in the Supporting Information^2^.
6. This dataset, whilst using complementary bibliometric data offered by Scopus through its analysis function (e.g., subject index area), was then analyzed through established bibliometric areas such as article characteristics, journals, citations, countries, authorship, institutions, and author keywords (Sweileh, 2018) to better understand the landscape of research on transgender SRH and potential gaps. Generating insight into these areas predominately took place through the popular bibliometric network and visualization software VOSviewer and Biblioshiny. VOSViewer is an open source and freely downloadable software (Van Eck & Waltman, 2021) that can generate network visualizations from Scopus bibliometric data. This aided in creating network maps around author keywords, author citations, and countries. It also offers a thesaurus function that can remove unwanted keywords or combine synonymous words together to reduce the “noise” in mapping and so produce a clearer figure. A record of thesaurus words used can be found in the Supporting Information and exported from Excel into a text file document for use within VOSViewer. Using RStudios (RStudio, 2021) and the Bibliometrix (Aria & Cuccurullo, 2017) R library package, Biblioshiny was also used to produce further bibliometric data insights. These include a three-fields plot (e.g., a figure showing the relationship between three data fields) and a country collaboration map (e.g., a map of countries by their productivity and relationships of collaborating corresponding authors). Where needed, analysis was also tabulated directly within Excel upon the dataset obtained from Scopus to contribute to this (e.g., reviewing the growth of document publications, etc.).

### Limitations to bibliometric analysis

The utilized software for bibliometric data analysis is limited to tackling a singular literature database at a time (AlRyalat et al., 2019), which could limit the inclusion of articles. Using Scopus as a large, multidisciplinary article database helps overcome this limitation, however, it can still have gaps, especially in retrieval of articles in non-English and non-Latin alphabet-based languages. Added barriers also include limitations inherent in search databases. For example, geographic information for the authors is captured as metadata, but not the country/geographical site where the study being reported was conducted. Issues also arise around the differential formatting of author abstracts and other data fields that makes automatic retrieval of information difficult. To address these limitations, an additional content analysis was decided upon to further our understanding of the retrieved literature and complement the bibliometric analysis.

### Content analysis

A content analysis was utilized to code for information not present within the retrieved bibliometric data (Coronado, Wurtzel, Simon, Riddle, & George, 2011). This was achieved by:

1. Reviewing each data entry’s title and abstract to code for the article’s scope of inclusion (e.g., what identities of the transgender community they engaged with) and the geographical site of the research.
2. These initial codes were then reviewed and finalized to create a consistent dataset for quantitative analysis using Excel.

## Results

### Control search

Preliminary literature searches and review revealed that the United States dominated heavily within the field of transgender health research. To understand if this was also the case for SRH, a control literature search on “sexual and reproductive health” within Scopus was carried out that retrieved over 12,000 documents, 32% of which were published from US authors. From this work’s search strategy (see Supporting Information) 914 articles were retrieved, with 41% published from US authors – highlighting that the USA’s high authorship productivity within trans SRH literature is comparable to levels found in transgender health and in SRH literature.

### Characteristics

**Figure 1.**
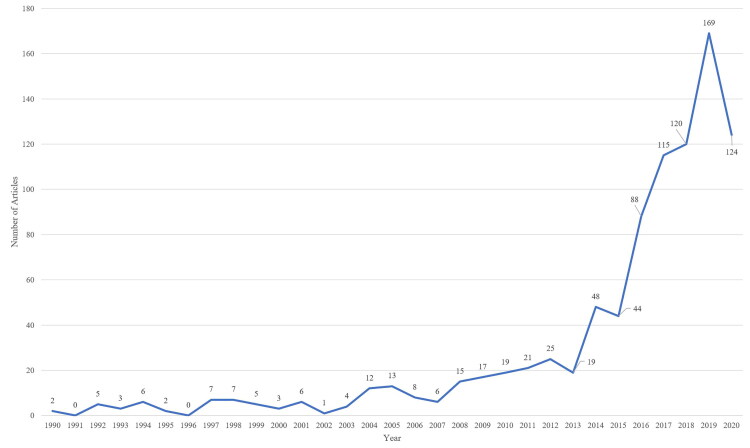
A line chart of retrieved publication numbers per year.

From the finalized dataset, 914 peer-reviewed articles were retrieved that covered a time span of 30 years between 1990 and 2020. English was the most frequently used language (884; 96.7%) followed by Portuguese (10; 1.1%), and Spanish (9; 1.0%). Retrieved from within 274 journals, 799 (87.4%) of these articles were indexed within medicine (see Table 1), 201 in social sciences (22.0%), and 151 in psychology (19.5%). Growth in articles (see Figure 1) was limited until 2004, increasing incrementally from here and experience sustained growth from 2013.

**Table 1.** Top indexed journal sources.

Indexed subject area	**Number of articles**	% (*n* = 914)
Medicine	799	87.4
Social Sciences	201	22.0
Psychology	151	19.5
Immunology and Microbiology	96	10.5
Arts and Humanities	43	4.7
Multidisciplinary	11	1.2
Pharmacology, Toxicology and Pharmaceutics	10	1.1
Other	13	1.3

Note: As articles are indexed in more than one subject area, the number of articles exceeds 100%.

### Most active journals and issue focus

Six of the top 10 journals (see Table 2) focused on HIV (Human Immunodeficiency Virus)/other Sexual Transmitted Infection (STI), followed by transgender health, “transgenderism,” LGBT health, and a journal on culture, health, and sexuality. The AIDS and Behavior journal held the highest citation count whilst the International Journal of Transgenderism had the highest citation per article (27.8). All top 10 journals were situated in high income countries: United States (4), Canada (2), UK (2), Australia (1), and the Netherlands (1). Together, articles from the top 10 journals account for 37.2% (340 articles) of the total literature retrieved.

**Table 2.** Top 10 producing journals.

Name of journal	Total	% (*n* = 914)	C	C/A	**Country** ^a^
AIDS and Behavior	91	10.0	1541	16.9	Netherlands
AIDS Care – Psychological and socio-medical aspects of AIDS/ HIV	41	4.5	1065	26.0	UK
Journal of the International AIDS Society	34	3.7	329	9.7	Canada
International Journal of STD and AIDS	31	3.2	312	10.1	UK
Transgender Health	27	3.0	129	4.8	United States
International Journal of Transgenderism	26	2.8	724	27.8	United States
Archives of Sexual Behavior	26	2.8	521	20.0	Canada
LGBT Health	24	2.6	387	16.1	United States
AIDS Education and Prevention	20	2.2	374	18.7	United States
Culture, Health and Sexuality	20	2.2	432	21.6	Australia

a Determined by the editor’s institutional location.

### Citation analysis

**Figure 2.**
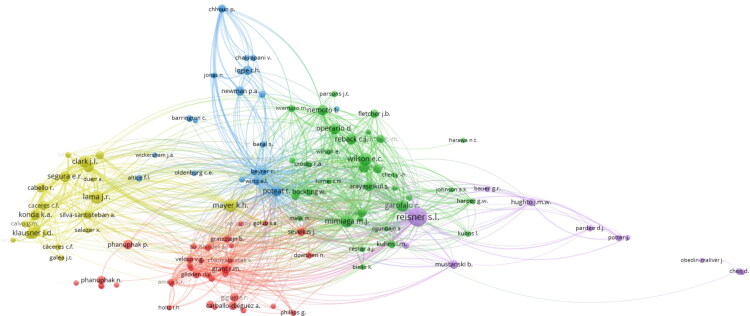
A citation by author network analysis that highlights authors who were cited together within the retrieved literature. The five colors represent clustering analysis by VOSviewer, outlining related groups of authors in this instance. Authors with less than five documents were excluded to improve visibility within the figure. Created with VOSviewer.

Retrieved articles received a collective 17,377 citations, ranging from a minimum of 0 to a maximum of 641 article citations with a mean of 22.7 and median of 9.5 citations per article. Table 3 shows the top 10 cited articles, the highest coming from “The Lancet Infectious Diseases” journal involving a systematic review and meta-analysis of studies between 2000 and 2011 around HIV burden in transgender women. Figure 2 outlines a citation by author network that shows the relations of authors based on the frequency of their citation with one another. Five distinct clusters are illustrated with Reisner holding the highest link strength (1478) in terms of their citation relation by other authors.

**Table 3.** Top 10 cited articles.

Title	Year	Source	C
Worldwide burden of HIV in transgender women: A systematic review and meta-analysis	2013	The Lancet Infectious Diseases	641
Uptake of pre-exposure prophylaxis, sexual practices, and HIV incidence in men and transgender women who have sex with men: A cohort study	2014	The Lancet Infectious Diseases	555
Overlooked, misunderstood and at-risk: Exploring the lives and HIV risk of ethnic minority male-to-female transgender youth	2006	Journal of Adolescent Health	322
Transgender health: Findings from two needs assessment studies in Philadelphia	2005	Health and Social Work	296
Managing uncertainty: A grounded theory of stigma in transgender health care encounters	2013	Social Science and Medicine	247
Transgender HIV prevention: A qualitative needs assessment	1998	AIDS Care - Psychological and Socio-Medical Aspects of AIDS/HIV	210
Health care utilization, barriers to care, and hormone usage among male-to-female transgender persons in New York City	2009	American Journal of Public Health	205
HIV risk behaviors among male-to-female transgender persons of color in San Francisco	2004	American Journal of Public Health	197
Gender Affirmation: A Framework for Conceptualizing Risk Behavior Among Transgender Women of Color	2013	Sex Roles	185
From efficacy to effectiveness: Facilitators and barriers to PrEP acceptability and motivations for adherence among MSM and transgender women in New York City	2013	AIDS Patient Care and STDs	185

### Geographic analysis

**Figure 3.**
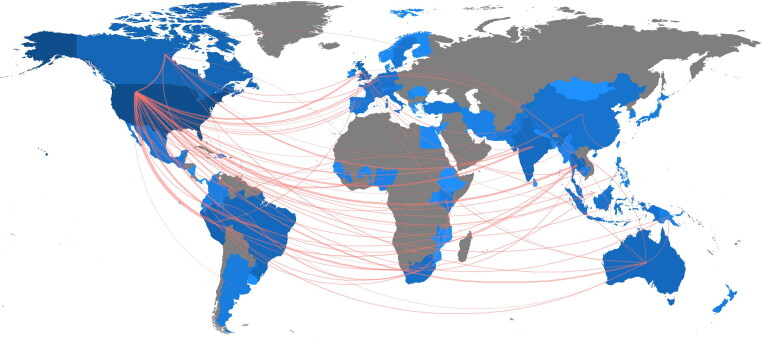
Country collaboration map of authors. This map shows international author collaboration through the orange lines between countries, showing activity highly centered between the United States and other countries. The spectrum of blue shading is indicative of document production with darker hues highlighting greater productivity and lighter hues lower productivity (see Table 4). Created with Biblioshiny.

**Figure 4.**
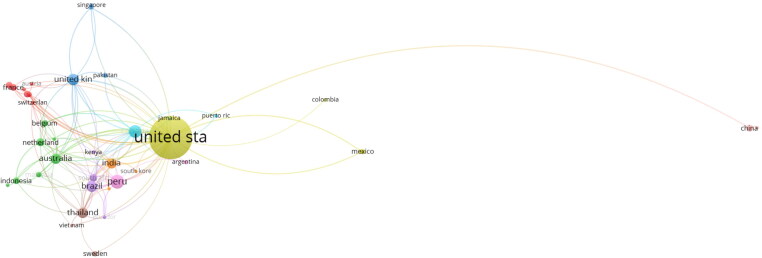
A coauthorship by country network analysis that outlines the relationship of authors based on them being coauthors within the retrieved documents. The United States is the largest interconnecting node between country authors. The nine colors represent clustering analysis by VOSviewer, outlining related international author collaborations in this instance. Created with VOSviewer.

There were 3653 authors found across 46 affiliated countries, the highest publication volume (see Table 4) seen in the United States with 395 articles (43.2%) followed by Brazil (20; 2.2%), and Canada (20; 2.2%). Figure 3 outlines intercountry collaborations and Figure 4 visualizes the retrieved author network, both highlighting the strong connections with the United States. In Figure 4, China and Columbia can be seen to only have collaborative connections with the United States and themselves, compared to the multi-country collaborations of the other countries present.

**Table 4.** Top 10 active countries by corresponding authorship.

Country	Articles (*n* = 914)	%
United States	395	43.2
Brazil	20	2.2
Canada	20	2.2
India	14	1.5
UK	11	1.2
Australia	10	1.1
Belgium	9	1.0
Italy	9	1.0
Peru	9	1.0
Netherlands	8	0.9

### Active authorship and institutions

**Figure 5.**
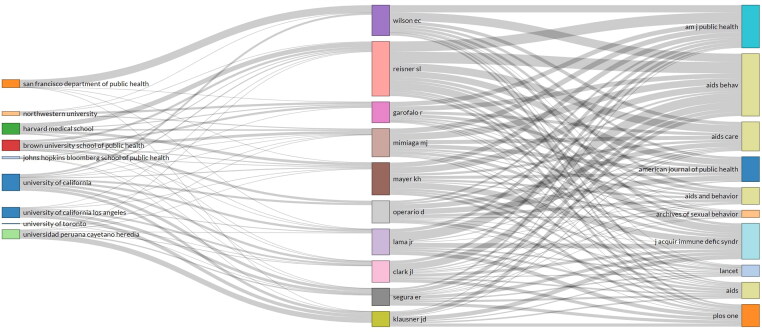
A three-fields plot that shows the relationship of authors between their institutions and cited sources within the retrieved literature. Created with Biblioshiny.

Of the 3635 authors, 47 of these published single-authored documents and 3588 published multiple-authored documents where there was an average of four authors per article, showing high levels of collaborative authorship in the documents retrieved. Table 5 shows the top 10 active authors within the articles retrieved; eight authors affiliated with United States institutions and two Peruvian institutions. Dr. Reisner, S.L. ranking the highest with 57 articles (6.2%) who specializes in transgender health and epidemiology of HIV, and other STIs. Seen in Table 6, US institutions were the most active, with the University of California ranking the highest with 3 non-US institutions present in the top 10. Figure 5 presents a visualization of authors compared with their affiliations and journal sources.

**Table 5.** Top 10 active authors.

Name	Total articles	% (*n* = 914)	**Affiliation** ^a^
Reisner SL	57	6.2	Fenway Institute, Boston, USA
Lama JR	26	2.8	Center for AIDS Prevention Studies (CAPS), AIDS Research Institute, University of California, USA
Wilson EC	26	2.8	Asociación Civil Impacta Salud y Educación, Lima, Peru
Mayer KH	25	2.7	Fenway Institute, Boston, USA
Mimiaga MJ	24	2.6	Fenway Institute, Boston, USA
Garofalo R	21	2.3	Children’s Memorial Hospital, Division of Adolescent Medicine, Chicago, USA
Clark JL	20	2.2	Department of Medicine, Division of Infectious Diseases, University of California Los Angeles, USA
Segura ER	19	2.1	Universidad Peruana Cayetano Heredia, Lima, Peru
Klausner JD	18	2.0	Division of Infectious Diseases, Department of Medicine, University of California, Los Angeles, USA
Operario D	18	2.0	Department of Behavior and Social Sciences, Brown University, Providence, USA

a Obtained through their Scopus/Google Scholar author profile.

**Table 6.** Top 10 active institutions.

Institution	Total articles	% (*n* = 914)	Country
University of California	296	29.6	United States
Johns Hopkins Bloomberg School of Public Health	84	8.4	United States
San Francisco Department of Public Health	83	8.3	United States
Harvard Medical School	67	6.7	United States
Northwestern University	67	6.7	United States
University of Toronto	59	5.9	Canada
Universidad Peruana Cayetano Heredia	58	5.8	Peru
Brown University School of Public Health	54	5.4	United States
Thai Red Cross Aids Research Center	52	5.2	Thailand
University of Washington	40	4.0	United States

### Author keywords

**Figure 6.**
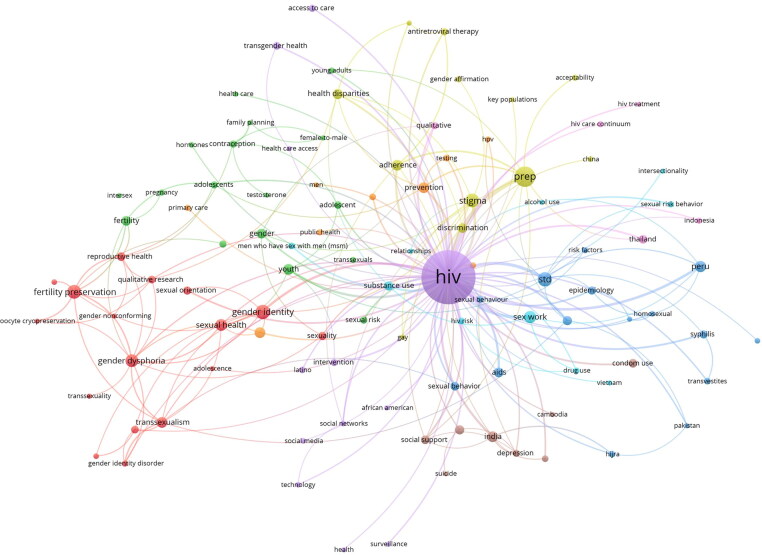
A co-occurrence analysis of author keywords that shows the joint use of keywords amongst the authors from the retrieved literature. Keywords that occurred less than five times were excluded and the remaining filtered with the VOSviewer thesaurus function (see additional materials) to reduce noise (e.g., crowding) within the figure. Created with VOSviewer.

Literature retrieved contained 1399 author keywords from the 914 articles. From this, 102 filtered keywords (those that have occurred at least a minimum of 5 times throughout the retrieved articles) are depicted in Figure 6. The visualization highlights 9 clusters that predominantly have links to HIV. The top occurring keywords included HIV, Pre-exposure Prophylaxis (PrEP), gender identity, stigma, and gender dysphoria.

## Content analysis

Patterns seen through the above bibliometric analysis focus on indexed data captured from Scopus. However, data such as where the study was conducted or what the population focus is, are not captured in bibliometric data throughout journal databases. To add further insight into this, a content analysis was undertaken on the retrieved document’s title and abstracts.

### Scope of inclusion

**Figure 7.**
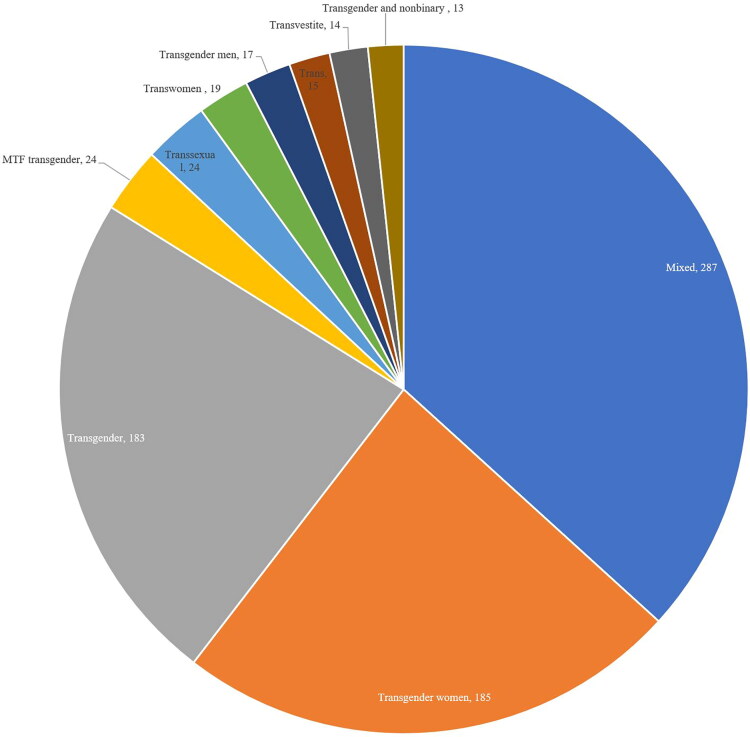
A pie chart of the top 10 coded terms for transgender inclusion within the articles’ title and abstract.

The population focus of the studies were coded for. This found studies largely holding a mixed population focus, e.g., including both cisgender and transgender people, in their research (287; 31.4%). As seen in Figure 7, coding for transgender identities found articles mostly focused on transgender women (185; 20%) and transgender (183; 20%). Table 7 highlights the varied nature of how transgender people are included within the literature, showing both the spectrum of variations on transgender, culturally specific language to engage with the trans community, but also the clinical and derogative language that was seen within the author keyword analysis.

**Table 7.** Scope of transgender inclusion.

Scope of inclusion	Count	% (*n* = 914)
Mixed^a^	287	31.4
Transgender women	185	20.2
Transgender	183	20.0
MTF transgender	24	2.6
Transsexual	24	2.6
Transwomen	19	2.1
Transgender men	17	1.9
Trans	15	1.6
Transvestite	14	1.5
Transgender and nonbinary	13	1.4
Hijra	10	1.1
Transgender and gender diverse	10	1.1
Intersex	9	1.0
Waria	9	1.0
FTM transgender	8	0.9
MTF transsexual	8	0.9
Transgendered	8	0.9
Hermaphrodite	7	0.8
Transgender and nonconforming	6	0.7
FTM transsexual	5	0.5
Trans women	4	0.4
MTF	3	0.3
MTF transgenders	3	0.3
Trans men	3	0.3
Transgender and gender expansive	3	0.3
Cannot discern	2	0.2
Kathoey	2	0.2
MTF and FTM	2	0.2
Transexual	2	0.2
Transgender women and transfeminine nonbinary	2	0.2
Two-spirit	2	0.2
Fa’afafine	1	0.1
FTM	1	0.1

**Table 7.** (*cont.*)

Scope of inclusion	Count	% (*n* = 914)
Gender diverse	1	0.1
Gender variant	1	0.1
Jogappas	1	0.1
Kathoeys and Toms	1	0.1
Kothi	1	0.1
Mahu	1	0.1
Mahuwahine	1	0.1
Mak nyah	1	0.1
Mak nyahs	1	0.1
MTF transgendered	1	0.1
Nonbinary	1	0.1
Queer and trans	1	0.1
Trans feminine	1	0.1
Transfemale	1	0.1
Transgender and genderqueer	1	0.1
Transgender, nonbinary, and gender diverse	1	0.1
Transgender, nonbinary, and gender expansive	1	0.1
Transgendered women	1	0.1
Transgenderism	1	0.1
Transgenders	1	0.1
Trans-masculine	1	0.1
Transpeople	1	0.1

Note: 2 data entries were coded as “cannot discern” and were omitted from this table.

a Articles that involved both the trans community and cisgender people were coded as “mixed” to highlight the split interest of the study with non-transgender groups.

### Study area

**Figure 8.**
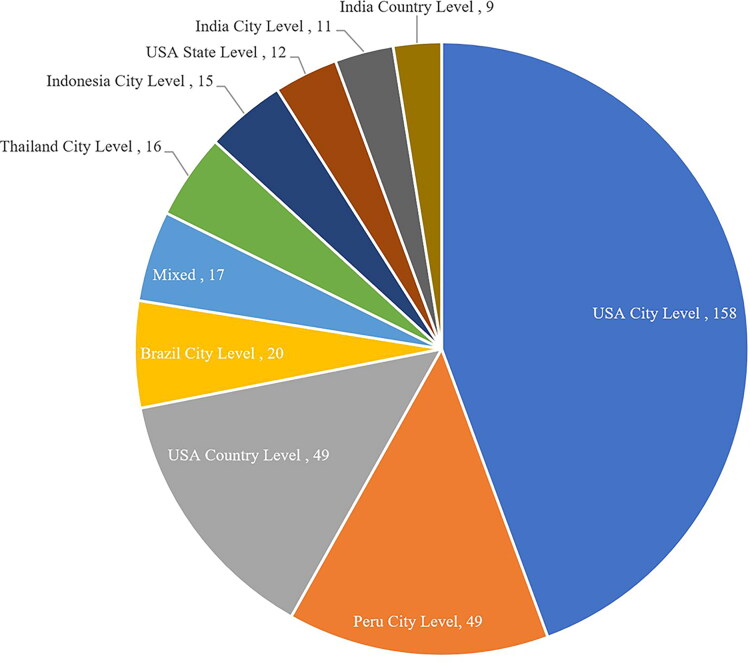
A pie chart of the top 10 coded areas of study within the articles’ title and abstract.

The areas of focus within the retrieved literature (see Table 8) centered on the United States at a city level (158; 17.3%), within Peru at a city level (49; 5.4%), and the United States at a country level (49; 5.4%). Figure 8 outlines the top 10 study areas, which includes Brazil, Thailand, Indonesia, and India at various levels of focus predominately with studies situated at the city level.

**Table 8.** Research study areas.

Areas of study	Count	% (*n* = 914)
USA City Level	158	17.3
Peru City Level	49	5.4
USA Country Level	49	5.4
Brazil City Level	20	2.2
Mixed^a^	17	1.9
Thailand City Level	16	1.8
Indonesia City Level	15	1.6
USA State Level	12	1.3
India City Level	11	1.2
India Country Level	9	1.0
India State Level	9	1.0
China City Level	8	0.9
Australia Country Level	6	0.7
Brazil Country Level	6	0.7
Guatemala City Level	6	0.7
Italy City Level	6	0.7
Malaysia City Level	6	0.7
Mexico City Level	6	0.7
Pakistan City Level	6	0.7
Argentina Country Level	5	0.5
Netherlands City Level	5	0.5
Peru Country Level	5	0.5
Spain City Level	5	0.5
Thailand Country Level	5	0.5
UK Country Level	5	0.5
USA Regional Level	5	0.5
Vietnam City Level	5	0.5
Cambodia City Level	4	0.4
Canada City Level	4	0.4
Canada Province Level	4	0.4
International	4	0.4
Jamaica City Level	4	0.4
Papua New Guinea City Level	4	0.4
Philippines City Level	4	0.4

**Table 8.** (*cont.*)

Areas of study	Count	% (*n* = 914)
Thailand Province Level	1	0.1
Thailand Regional Level	1	0.1
Timor-Leste Region Level	1	0.1
Turkey Country Level	1	0.1
Uganda City Level	1	0.1
UK City Level	1	0.1
Uruguay City Level	1	0.1
USA First People Level	1	0.1
Vanuatu City Level	1	0.1

Note: 309 data entries were coded as “cannot discern” and were omitted from this table.

a Articles that were based in multiple areas were coded as “mixed.”

**Table 8.** (*cont.*)

Areas of study	Count	% (*n* = 914)
Philippines City Level	4	0.4
USA Federal District Level	4	0.4
USA Territory Level	4	0.4
Australia City Level	3	0.3
Brazil Region Level	3	0.3
Cambodia Country Level	3	0.3
Canada Country Level	3	0.3
El Salvador City Level	3	0.3
India Regional Level	3	0.3
Myanmar City Level	3	0.3
Nigeria City Level	3	0.3
South Africa Province Level	3	0.3
Bangladesh City Level	2	0.2
Brazil State Level	2	0.2
China Country Level	2	0.2
Colombia City Level	2	0.2
Colombia Country Level	2	0.2
Dominican Republic Country Level	2	0.2
Ecuador City Level	2	0.2
Germany Country Level	2	0.2
Global	2	0.2
Iran City Level	2	0.2
Israel City Level	2	0.2
Jamaica Country Level	2	0.2
Lebanon City Level	2	0.2
Pakistan Country Level	2	0.2
Singapore City Level	2	0.2
South Africa City Level	2	0.2
Sub-Saharan Africa	2	0.2
Sweden City Level	2	0.2
Sweden Country Level	2	0.2
UK Nation Level	2	0.2
USA Country and Territory Level	2	0.2

**Table 8.** (*cont.*)

Areas of study	Count	% (*n* = 914)
USA State and Territory Level	2	0.2
Africa	1	0.1
Australia Regional Level	1	0.1
Australia State Level	1	0.1
Bangladesh Country Level	1	0.1
Belgium City Level	1	0.1
Belgium Country Level	1	0.1
Canada Region Level	1	0.1
China Province Level	1	0.1
Côte d’Ivoire Country Level	1	0.1
Egypt Country Level	1	0.1
Eswatini Country Level	1	0.1
Europe	1	0.1
Finland Country Level	1	0.1
Germany City Level	1	0.1
Greece City Level	1	0.1
Haiti Country Level	1	0.1
Hong Kong City Level	1	0.1
Italy Country Level	1	0.1
Kenya City Level	1	0.1
Malaysia Country Level	1	0.1
Mongolia City Level	1	0.1
Nepal City Level	1	0.1
Nepal Country Level	1	0.1
New Zealand (Aotearoa) Country Level	1	0.1
New Zealand City Level	1	0.1
Nigeria Country Level	1	0.1
Online	1	0.1
Pakistan Province Level	1	0.1
Portugal Country Level	1	0.1
Samoa	1	0.1
Serbia City Level	1	0.1
Southeast Asia	1	0.1

## Discussion

The above work undertook a bibliometric and content analysis on literature retrieved around transgender SRH. Key findings from these centered on three areas: (i) the United States as both a predominate site of publication as well as a geographical site of research being reported, (ii) clinical and disease-orientation of trans SRH issues studied, and (iii) continued use of derogative language.

Findings suggest academic engagement with trans SRH lags in comparison to wider transgender health research. Compared to articles retrieved by Sweileh (2018) in their bibliometric analysis of transgender health, this specific subarea of transgender SRH occupies a smaller body of articles. As identified in Figure 1 Transgender SRH literature experienced limited growth between 1990 and 2003, with 2004 seeing double figure growth and sustained growth in publications taking place from 2013 onward. As Sweileh outlines double figure growth in 1953 and sustained growth from 1993 onward for transgender health literature (p. 4) suggests a lag in scholarly interest around transgender SRH.

As seen within the control search the United States dominates research on transgender SRH in terms of authorship. Additional analysis undertaken into this showed that the study areas of the literature also have a high focus within the United States, specifically at a city level. This presents difficulties in extending these studies into applications elsewhere as both the voice of the author(s) and the trans community involved are within a United States context. This high presence of the United States throughout top producing institutions and authors within the retrieved documents also raises concern over Northern bias in the knowledge production within SRH and trans health scholarship. Content analysis also revealed that studies – both in the United States and internationally – tended to focus primarily on large metropolitan cities (e.g., New York or Lima) that leaves non-urban trans communities lacking in representation within SRH research.

Although predominant, the United States and European countries are not the sole contributors of knowledge on trans SRH. Despite the limitation of capturing non-indexed journals published in low-to-middle-income (LMI) countries in databases like Scopus, articles from 46 countries were retrieved. Table 4 outlining the inclusion of Brazil, India, and Peru following the United States, Canada, and European countries as top producing areas. Countries within Africa, Eastern Europe, and the Middle East had limited retrieval.

Keyword and content analysis highlighted that research centered heavily on sexual health diseases, mainly on HIV, HIV prevention and STIs in general that was further highlighted in the focal areas of top journals publishing this research (see Table 2). Outlining focuses of research centered around disease rather than dealing with trans SRH through rights affirming concepts. This table along with the top cited articles in Table 3 outlined journals coming from a clinical and biomedical specialization. This suggests a continued dominance of biomedical and clinical perspectives, to the neglect of key rights areas and risk of the medicalization of trans SRH (Johnson, 2019). Limited engagement with reproductive justice or other sexual and reproductive health rights (SRHR) issues such as violence, health socio-politics, reproductive rights, service access denial, etc. hinders emancipatory efforts (Schaaf et al., 2021) to overcome obstacles faced by the transgender community.

Throughout the retrieved articles dated language perceived as derogatory was utilized for referring to the transgender community with author keywords including terms such as “transsexualism” (19 occurrences), “transgendered persons” (10 occurrences), and “transgenderism” (6 occurrences) were present. These terms have been criticized for their pathologizing tendencies that present gender and sexual identities as mental disorders (Inch, 2016). The continued use of these within author keywords calls for raising awareness to explore/adapt to non-pathologizing language among researchers as well as academic publishers. Creating space to question the inclusion of these keywords within submissions and the need for more gender sensitive and inclusive indexing; the need for greater transgender awareness and reflection within academia and academic publishing is apparent.

The high number of articles and journals linked with medicine (see Tables 1 and 2), issues with language sensitivity (Bouman et al., 2017), and focus on disease in author keywords (see Figure 6) outlines a psycho-medicalization (Castro-Peraza et al., 2019) of the trans community. That instead of a holistic body of work on trans well-being and efforts to strive toward health justice, a fragmented literature exists that partitions transgender health into specific “conditions,” of pathologization and medical stratification. The content analysis on transgender inclusion within the articles reinforces this, revealing a disproportionate focus on certain groups within the transgender community, particularly transgender women (see Table 7). Further work is needed to understand and engage with the intersecting injustices and inequities that persist not only for certain trans identities, but across differing SRH contexts and impacts throughout the trans community so no one is left behind (Kapilashrami, 2020) in the UN’s SDGs aim for universal sexual and reproductive services by 2030.

The unique SRH contexts experienced across the trans spectrum implores the need to be inclusive of differential needs of this diverse group. Health issues and needs of trans men around fertility preservation of oocytes to non-binary trans people’s ability to access SRH care are often missed in trans health studies that continue to adopt binary concepts of male and female reproduction. This limits the current body of knowledge and demands more inclusive and expansive scopes to fully encompass the broad spectrum of the transgender community.

## Conclusion

This analysis shows recent growth in transgender SRH scholarship since 2014. However, literature sat predominately indexed in medical journals with top keywords and top journals publishing knowledge on trans SRH to be clinically focused. Limited engagement with reproductive justice, the broader spectrum of SRH rights, and the barriers faced by the trans community in realizing these highlights a need for further research within this area. Focus on article production within the United States, both in terms of authorship and as a geographical focus of study, creates a siloed body of knowledge. Engaging with greater international collaboration, supporting research by authors outside of the Western publishing sphere, and diversifying the study areas within research, particularly non-urban areas, would help contribute to widening and deepening the evidence base. Continuing use of derogative and outdated language to describe the transgender community outlines a need for further work around transgender awareness both among researchers and the research publishing process. Lastly, further need to engage with the full spectrum of trans identities within research is needed to better include the unique SRH contexts experienced throughout the community.

## Footnotes

1 For example, searching for “two-spirits” might ignore variations of its spelling. Adding a “*” wildcard allows spelling variations from the last t onward (e.g. two-spirits, two-spirited, two-spirit, etc.), ensuring that word variants throughout the documents are accounted for.

2 As search databases like Scopus change overtime, the process they go about retrieving article information can also change which may cause differences between the archived retrieved bibliometric dataset and data retrieved if using the search strategy directly within Scopus itself.

## Abbreviations

HIV: Human Immunodeficiency Virus
LMI/LMIC: low-to-middle-income countr(y/ies)
PrEP: pre-exposure prophylaxis
SDGs: sustainable development goals
STI/STIs: sexually transmitted infection(s)
SRH: sexual and reproductive health
SRHR: sexual and reproductive health rights
UN: United Nations
